# Loss of the Keratin Cytoskeleton Is Not Sufficient to Induce Epithelial Mesenchymal Transition in a Novel KRAS Driven Sporadic Lung Cancer Mouse Model

**DOI:** 10.1371/journal.pone.0057996

**Published:** 2013-03-11

**Authors:** Katharina König, Lydia Meder, Cornelia Kröger, Linda Diehl, Alexandra Florin, Ursula Rommerscheidt-Fuss, Philip Kahl, Eva Wardelmann, Thomas M. Magin, Reinhard Buettner, Lukas C. Heukamp

**Affiliations:** 1 Institute of Pathology, University of Cologne, Cologne, Germany; 2 Whitehead Institute of Biomedical Research, Cambridge, Massachusetts, United States of America; 3 Institutes of Molecular Medicine and Experimental Immunology, University of Bonn, Bonn, Germany; 4 Translational Centre for Regenerative Medicine and Institute of Biology, University of Leipzig, Leipzig, Germany; University of Melbourne, Australia

## Abstract

Epithelial-to-mesenchymal transition (EMT), the phenotypical change of cells from an epithelial to a mesenchymal type, is thought to be a key event in invasion and metastasis of adenocarcinomas. These changes involve loss of keratin expression as well as loss of cell polarity and adhesion. We here aimed to determine whether the loss of keratin expression itself drives increased invasion and metastasis in adenocarcinomas and whether keratin loss leads to the phenotypic changes associated with EMT. Therefore, we employed a recently described murine model in which conditional deletion of the Keratin cluster II by Cre-recombinase leads to the loss of the entire keratinmultiprotein family. These mice were crossed into a newly generated Cre-recombinase inducible KRAS-driven murine lung cancer model to examine the effect of keratin loss on morphology, invasion and metastasis as well as expression of EMT related genes in the resulting tumors. We here clearly show that loss of a functional keratin cytoskeleton did not significantly alter tumor morphology or biology in terms of invasion, metastasis, proliferation or tumor burden and did not lead to induction of EMT. Further, tumor cells did not induce synchronously expression of vimentin, which is often seen in EMT, to compensate for keratin loss. In summary, our data suggest that changes in cell shape and migration that underlie EMT are dependent on changes in signaling pathways that cause secondary changes in keratin expression and organization. Thus, we conclude that loss of the keratin cytoskeleton per se is not sufficient to causally drive EMT in this tumor model.

## Introduction

Phenotypical changes from an epithelial to a mesenchymal cell type, referred to as epithelial-to-mesenchymal transition (EMT), are key steps to invasion and metastasis of cancer. One hallmark of EMT is thought to be remodeling of the cytoskeleton, such as down regulation of epithelial keratins, which leads to alterations in cell-to-cell adhesions and changes in polarity and cell motility [1]. These changes have been described in most types of adenocarcinomas and are believed to support invasiveness of tumors and metastasis formation [2] and resistance to chemotherapy [3].

Lung cancer, the most common cause of cancer deaths worldwide, has traditionally been divided into small-cell lung carcinomas (SCLC) and non-small-cell lung carcinomas (NSCLC). SCLCs make up 20% of lung cancers [4]. NSCLCs are further subdivided into three histological subtypes: squamous cell carcinoma, adenocarcinoma and neuroendocrine tumors. NSCLCs form large cohesive primary tumors that differ from SCLCs, which are clinically very aggressive and reveal a mortality rate of 95% [5]. SCLCs metastasize very early, are non-cohesive and show a diffuse and infiltrative growth pattern. Histologically, they are characterized by an incomplete and condensed keratin cytoskeleton displaying a punctuated and perinuclear distribution.

With the increasing application of targeted EGFR tyrosine kinase inhibitor (TKI) therapy in adenocarcinomas of the lung, several resistance mechanisms against this therapy have emerged. On the one hand, mutations causing steric changes in the target protein or evasion of the inhibited target via the recruitment of a second receptor or signal transducer have been observed. On the other hand, lineage transformation by undergoing epithelial-to-mesenchymal transition (EMT) [6] or the relapse of TKI treated NSCLS as small cell lung cancer (SCLC) have been described [7], [8]. Thus, these clinical observations support the hypothesis that progression from NSCLCs to SCLCs may be triggered by EMT.

Since loss of keratin expression is thought to be central to EMT, detecting reduced or lost expression of keratins *in vivo* is used as a marker of EMT in many *in vivo* model systems as well as on histological human sections. Loss of keratins leads to loss of cell junctions, such as functional desmosomes and tights junctions, and therefore strong cell-cell adhesion [9], [10]. In particular, the expression of adherens junctions is regulated by EMT genes both in adenocarcinomas and squamous cell carcinomas of the lung [11].

However, the precise functional role of keratin loss on tumor growth, metastasis and invasion and its role in EMT is not clearly established in any tumor model. We therefore investigated whether complete loss of keratin expression induces EMT, invasion and metastasis. To this end, we developed a novel inducible mouse model for lung adenocarcinoma defective in the entire keratin type II cluster causing failure to form any functional keratin filaments. Using this model, we here show that complete keratin loss in KRAS mutated lung tumors does not affect tumor morphology, invasion or metastasis, indicating that loss of the keratin cytoskeleton is not driving the transition of lung tumors into small cell lung carcinoma or a sarcomatoid phenotype. Furthermore, EMT markers were not up regulated in keratin-deficient adenocarcinomas.

## Materials and Methods

### Ethics Statement

This study was carried out in strict accordance with the recommendations of the FELASA. The protocol was approved by the Committee on the Ethics of Animal Experiments of the University of Bonn. All efforts were made to minimize suffering.

### Generation of RASLO Transgenic Mice

**Figure 1:**
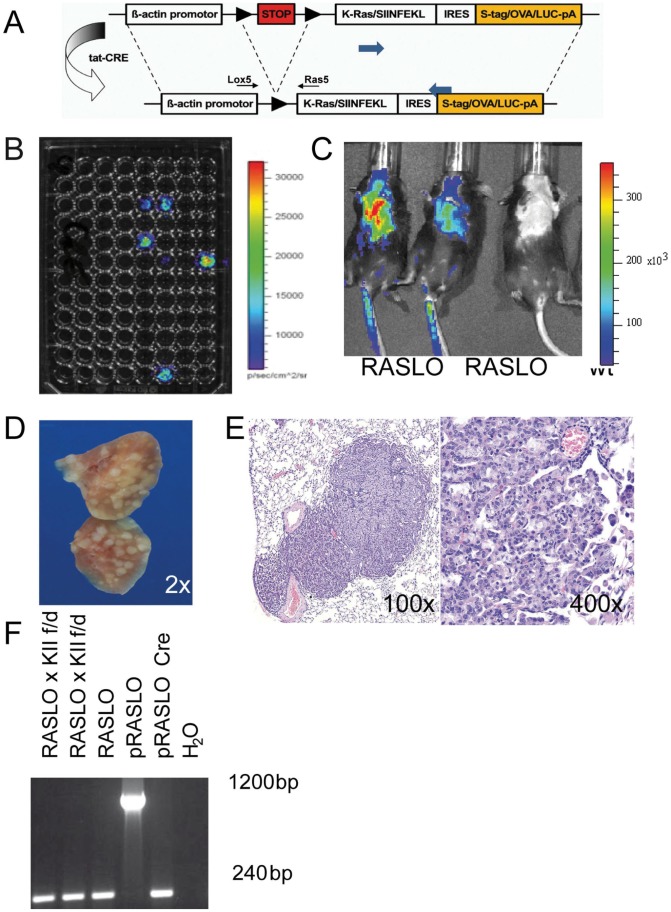
RASLO mice rapidly develop lung tumors after AdCre administration. (A) Schematic construct showing the strategy used to generate the inducible KRAS driven lung cancer model (RASLO). (B) Bio-Luminescence image taken with an IVIS-200 (Xenogen) camera of tail fibroblasts cultures of the RASLO founder mice after treatment with 2 µM TAT-CRE protein and addition of luciferin right before imaging, showing strong signals in 5 founder mice. (C) Luciferase imaging (IVIS-200) for 1 min at medium sensitivity of RASLO mice 6 weeks after induction with 2×10^7^ PFU of Adeno-CRE i.n.(+) or breeding controls (−). Mice were injected i.p. with luciferin in 200 µl PBS and anesthetized by isoflurane inhalation. (D) Macroscopic image of the lung of a RASLO mouse 6 weeks after of AdCre administration, and (E) HE stained section with multiple tumors at 100x and 400x magnification. (F) PCR analysis of DNA isolated from cell lines derived from tumor of RASLO and RASLO×KIIf/d mice show a PCR product of 240 bp indicating that the Stop cassette has been successfully removed by CRE recombinase in the tumor. The vector used to generate the mouse strain (pRASLO) prior to CRE treatment shows a 1200 bp fragment. The same vector following Cre treatment in vitro serves as a positive control for the CRE mediated excision and shows the expected 240 bp band after recombination.

To induce lung tumors, we generated an expression vector expressing a mutated KRAS^VAL12^ as well as a fusion molecule consisting of ovalbumin, an S-tag and a luciferase molecule driven by a 1.8-kb chicken ß-actin promoter [4] after Cre-mediated removal of a STOP codon. RASLO mice were generated by pronucleus injection of C57Bl/6×FVB F1 embryos with the oncogenic KRAS construct depicted in Figure 1A. Mice were bred in the central animal facility of the University of Bonn according to the Federation of European Laboratory Animal Science Association guidelines. The Animal Care Commission of Nordrhein-Westfalen approved all mouse experiments.

### Screening for RASLO Transgenic Mice

Tail clippings of founders were used to set up fibroblast cultures and PCR analysis. Therefore tail tips were sterilized with 70% Ethanol, cut into small pieces and digested with collagenase for four hours at 37°C. They were cultivated in DMEM medium supplied with 8% FCS, 2 mM glutamine and penicillin/streptomycin. After five days, tail fibroblasts were treated with 2 µM Tat-Cre protein for seven hours and the next day analyzed under the IVIS 200 bioluminescence camera after the addition of luciferin to the culture medium. PCR and luciferase assay positive founders were used to establish several RASLO strains. Genotyping was performed by tail PCR using forward (5′-CAGTGCAATGAGGGACCAGT-3′) and reverse primers (5′-CACCCTGTCTTGTCTTTGCTGATG-3′).

### Production and Administration of Adenoviral CRE

HEK 293 cells were infected with recombinant adenovirus expressing Cre-recombinase (AdCre) [12]with (MOI = 5). After five days, cells were harvested and virus was released by rapid thawing and freezing cycles. Virus was purified by ultracentrifugation on a cesium chloride gradient [13] Thereafter, virus stocks were generated via concentration using Slidalyzer cassettes (Thermo Fisher Scientific).

Prior to nasal application of AdCre mice were anesthetized with a combination of 10 mg/ml Ketamin/0.1% Rompun in PBS. Depending on the body weight of the mouse between 150 µl and 200 µl was injected i.p. 2×10^7^ PFU of AdCre was pipetted on the nose of the mice to be inhaled. Animals were observed until fully awake and comfortable. No signs of stress and discomfort were observed. After tumor induction, animals were monitored daily for signs of discomfort or respiratory distress. Mice were excluded for further analyses, when mice showed symptoms of respiratory stress. Mice were imaged on a regular basis under IVIS 200 bioluminescence camera (PerkinElmer). Briefly, mice were anesthetized by isoflurane inhalation and then received 2.8 mg D-Luciferin Firefly (Caliper) in 200 µl PBS i.p. and imaged on a heated stage. Mice were killed by cervical dislocation after 6 weeks of AdCre administration. Lungs were immediately fixed in 4% PBS-buffered formalin for 24 hours for paraffin embedding, or snap frozen in liquid nitrogen for cryo-preservation.

### Polymerase Chain Reaction (PCR)

DNA was isolated from cell lines generated from transgenic mouse lung tumors or from fresh frozen (FF) lung tissue with DNA Mini Kit (Qiagen). 50 ng of template DNA was used per reaction. Each PCR reaction contained 1x buffer, 0.2 mM dNTP, 2 mM MgCl_2_, 0.2 U Taq polymerase and 0.2 µM primer. To show successful excision of the loxP sites 5′ Lox5 forward: 5′-CTGCTAACCATGTTCATGCC-3′ and 3′ Ras5 reverse: 5′-CCTACGCCACAAGCTCCAAC-3′ were used yielding a product of 240 bp. Without excision of the STOP codon these primers yield a product of 1.2 kb. To confirm keratin locus excision the following primers were used: DEL (forward 5′- TGAACCCAGGAGGTTGAGAC-3′ and reverse 5′- TGGCGTCGTGATTAGTGATGA) and FLOX (forward 5′- GATAACCGTATTACCGCCTTTG-3′ and reverse 5′- CGCCCTCTTGTCTATATCAACC-3′).

### Cell Lines

The following cell lines of human origin were used: A549, H1975, H460, HCC827, DMS114, and SW1271 were obtained from American Type Culture Collection. R. Thomas (University of Cologne) kindly provided: N417 [14], PC9 [15]. GLC1, GLC2 [16], and GLC36 [17] were a kind gift of Dr. L. de Leij (University of Groningen, Groningen, The Netherlands). NSCLC and SCLC cell lines were cultivated in DMEM medium supplemented with 8% FCS incubated at 37°C and 5% CO_2_.

Mouse lung cell lines were generated from RASLO KII+/+ and KIIf/− mice. Tumors were excised with a scalpel and digested for at least 3 hours at 37°C in DMEM medium/Ham’s F12 Medium with L-glutamine supplemented with 2% bovine albumin, 0.5 µg/ml hydrocortisone, 600 U/ml collagenase, 5 µg/ml human insulin, 200 U/ml hyaluronidase, Hepes buffer 10 mM, and Penicillin/Streptomycin. The cell suspension was filtered twice and red blood cells lysed with ACK lysis buffer. Cells were cultivated in DMEM medium supplemented with murine EGF 20 ng/ml, basic Fibroblast Growth Factors (bFGF) 20 ng/ml, Heparin sodium salt from porcine intestinal mucosa 4 µg/ml, B-27 Serum-Free Supplement, Penicillin/Streptomycin and Gentamycin 35 µg/ml.

### qRT-PCR

Total RNA was isolated from human lung cancer cell lines or cryo-preserved mouse lung tissue (5 times 10 µm slides) by RNeasy Mini Kit (Qiagen). 500 ng of total RNA was transcribed into cDNA using SuperScript™ III H^−^ Reverse Transcriptase and Oligo(dt)12–18 Primer (Life Technologies). qRT-PCR was performed with SYBR Green (Qiagen) with primers directed against keratin 8 (forward 5′-GCCGTGGTTGTGAAGAAGA-3′ and reverse 5′-CTGTTCCCAGTGCTACCCT-3′). As a housekeeping control we used 28 s RNA (forward 5′-CCCAGTGCTCTGAATGTCAA-3′ and reverse 5′-AGTGGGAATCTCGTTCATCC-3′). Luciferase primers had the following sequence: forward 5′-TTGCATTTTGATCCAGTCGAG-3′ and reverse 5′- TCGAGAGCGTGGATCAAACG-3′; and as a housekeeping control 18 s: forward 5′- ACAGCCAGGTTCTGGCCAACGG-3′ and reverse 5′- TGACCGCGGACAGAAGGCCC-3′.

All qRT-PCRs were run on the 7900HT Fast Real-Time PCR System (Applied Biosystems), and analyzed with SDS2.2 software (Applied Biosystems). All samples were analyzed in triplicates and expression level was calculated using ΔΔCT method.

### Immunofluorescence of Lung Cancer Cell Lines

Cells were plated on cover slips overnight and fixed in ice-cold methanol for 5 minutes, then stained with pan-Keratin (1∶100, MNF116, Dako) and DP-1 (1∶100, DP-1, Progen) diluted in TBS/1%BSA/5%NGS for 1 hour at room temperature. Corresponding secondary antibodies goat anti-gp^Alexa488^ (1∶800, Life Technologies), goat anti-mouse^TexasRed^ (1∶800, Life Technologies) and DAPI (1∶1000, Life Technologies) diluted in TBS/1%BSA/5%NGS were added to the cells for 45 minutes at room temperature. Images were taken with an inverted microscope fitted with an ApoTome (Zeiss).

### Immunohistochemistry

Tissues were fixed in 4% PBS-buffered formalin, embedded in paraffin (FFPE). 3 µm slides were used for histological stains. Immunohistochemistry was performed as described previously [18]. The following antibodies were used: Ki67 (1∶25, TEC-3, Dako), K8/18 (1∶100, GP11, Progen), Vimentin (1∶100, EPR3776, Abcam), E-Cadherin (1∶50, SC-8426, Santa Cruz Biotechnology), ß-Catenin (1∶100, #4270, Cell Signaling), CD56 (1∶50, RNL-1, Abcam), Snail (1∶200, Abcam), Synaptophysin (1∶200, SY38, Abcam), chromogranin A (1∶200, abcam), Slug (1∶100, Abcam), pERK (1∶50, Cell Signaling), CD44 (1∶50, Abcam). All slides were scanned with a Pannoramic 250 slide scanner (3D Histech.com).

### Statistics

Statistics were calculated with Excel, Prism and SPSS. Error bars indicate standard error of the mean. The Student’s t-test was used to analyze data for significant differences. P-values <0.05 were regarded as significant and indicated in the figures as follows: *p≤ 0.05, **p≤0.01, ***p≤0.001.

## Results

### Generation of RASLO Murine Lung Cancer Model

We generated a novel transgenic murine model (RASLO) for lung adenocarcinoma, which upon CRE recombinase-induced removal of a stop codon is driven by a constitutively activated oncogenic KRAS^Val12^ under the control of a 1.8 kb chicken ß-actin promoter. The construct also expresses a fusion gene consisting of an S-tag (for immunohistochemistry), luciferase (for in vivo detection of tumors) and chicken ovalbumin (as a model tumor antigen) by means of a polyoma virus internal ribosomal re-entry site (**Fig. 1A**). RASLO mice were generated by pronucleus injection of C57Bl/6×FVB F1 embryos. The resulting animals were screened for successful integration of a functional construct, by incubation of tail fibroblast cultures with recombinant Tat-Cre protein [19] to excise the stop cassette and activate the construct *in vitro*. Subsequently, these fibroblasts were imaged with an IVIS 200 bioluminescence camera after addition of luciferin to the culture medium (**Fig. 1B**) to identify animals with a fully functional integration of the RASLO construct. Five founders were identified based on PCR and luciferase assays, one of which showed germ line transmission.

We applied an adenovirus expressing CRE recombinase (AdCre) intranasally (i.n.) to RASLO mice. Lung tumors developed in these mice after 6 to 8 weeks that could be visualized *in vivo* by detection of bioluminescence after i.p. injection of luciferin (**Fig. 1C**) and were macroscopically detectable *ex vivo* as numerous white nodules covering the lungs (**Fig. 1D**). Tumors arising in the RASLO mice treated with AdCre comprised multiple papillary adenomas and invasive papillary adenocarcinomas (**Fig. 1E**), which is consistent with KRAS driven lung cancer models described previously [4], [20]. Tumor formation was exclusively established in the lung, as we did not observe tumor formation in any other organ of the AdCre-treated RASLO mice. Genomic PCR analysis of cell lines established from RASLO lung tumors revealed successful removal of the stop cassette (**Fig. 1F**).

### Reduced Keratin Expression in SCLC

**Figure 2:**
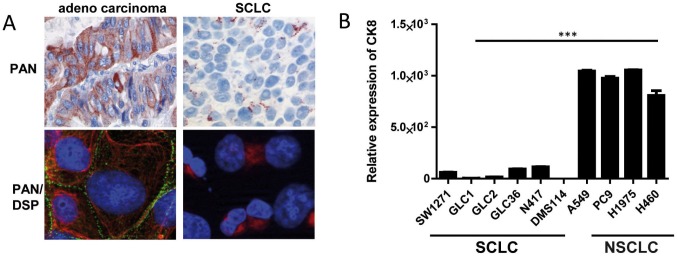
Human tumors samples and human NSCLC cell lines show higher expression levels of keratin compared to SCLC. (A) Immunohistochemistry of FFPE tumor samples for pan Keratin of adenocarcinoma of the lung and a typical SCLC (upper panels of A). Immunofluorescence of an adenocarcinoma (HCC827) and SCLC cell line (GLC36) stained for pan Keratin (red) and desmoplakin (DSP in green) (A, lower panels). (B) Total RNA was isolated from a panel of human SCLC and NSCLC cell lines. Relative mRNA expression of keratin 8 was measured by qRT-PCR Mean relative expression of quadruplicate analyzes for each cell lines are depicted and the SEM is indicated. Statistical significance was calculated using a Student’s t-test: *p≤ 0.05, **p≤0.01, ***p≤0.001. Representative of three independent experiments.

Loss of keratin expression is commonly observed during EMT as well as transformation from an adenocarcinoma to a small cell carcinoma (SCLC) [8]. Therefore, we stained human lung adenocarcinomas and small cell lung cancer specimens for keratins and observed only perinuclearly condensed keratin staining in the SCLC samples compared to strong cytoplasmic staining in the cohesively growing lung adenocarcinomas (**Fig. 2A** **upper panels**). A similar staining pattern of keratins was observed in cell cultures of adenocarcinoma and SCLC cell lines by immunofluorescence staining (**Fig. 2A** **, bottom panels**). Furthermore, we stained for desmoplakin that, when absent, leads to more invasive tumor growth and is absent or reduced in many human epithelial cancers [21]. In SCLC, desmoplakin was absent, whereas NSCLC expressed desmoplakin (**Fig. 2A**). We further investigated whether keratins are differentially expressed in NSCLC and SCLC cell lines. Indeed, SCLC cell lines expressed little or no mRNA for keratin 8, whereas NSCLC lines expressed high levels of keratin 8 mRNA (**Fig. 2B**).

### Keratin Loss in vivo does not Lead to EMT or a More Aggressive Tumor Biology

**Figure 3:**
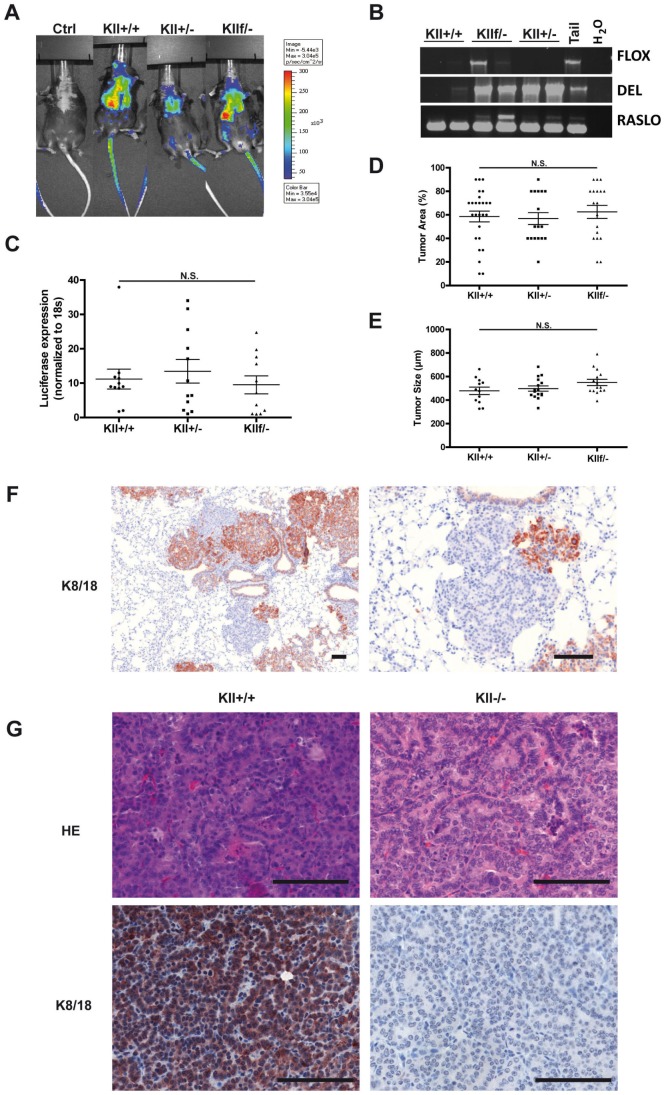
Tumors with In vivo inactivation of the Keratin type I cluster show no significant alteration in tumor biology. (A) Groups of RASLO transgenic mice crossed onto different Keratin II cluster alleles were anesthetized with Ketamine/Rompun, and 2×10^∧^7 pfu AdCre were administered i.n. six week previously. For tumor detection, mice were injected i.p. with luciferin in 200 µl PBS and imaged with an IVIS-200 (Xenogen) camera. Bioluminescense comparing representative wild-type (ctrl), RASLO transgenic with wild-type Keratin locus (KII +/+), RASLO transgenic with heterozygous Keratin cluster II expression (KII +/−) and RASLO transgenic mice with one knock-out and one floxed Keratin cluster II allele (KII f/−) are shown here. The different groups showed bioluminescence signals with comparable intensity. (B) Allele specific PCR analyses for DNA isolated from cryo-conserved lung tumors of RASLO mice for detecting the FLOX, DEL, and RASLO alleles were performed. (C) Luciferase mRNA expression level were determined in cryo-conserved lungs by qRT-PCR to estimate the tumor load. Expression level was normalized to 18 s RNA. (D) Quantification of tumor burden by area on three sections per lung. Three representative HE sections per lung were analyzed with respect to percentage of tumor area (upper panel) and tumor size (lower panel). FFPE sections were stained for K8/18 and imaged with Pannoramic250 (3DHistech) slide scanner. Maximal tumor diameter of up to thirty tumors representative tumor per slide was measured with the 3DHistech Pannoramic Viewer and the mean maximal diameter compare between the different genotypes. (F) Immunohistochemical staining for keratins 8 and 18 in tumors of RASLO KII f/− mice. (G). The scale bar represents 100 µm. Wild type RASLO tumors (left) compared to KII−/− tumors on the right are shown as HE stains (upper panels) and Keratin 8 and 18 (lower panels) values are depicted as mean +/− SEM. Statistical significance was calculated using a Student’s t-test: *p≤ 0.05, **p≤0.01, ***p≤0.001. Representative of three independent experiments.

To investigate whether loss of keratin expression in adenocarcinomas of the lung functionally leads to worse tumor biology by inducing EMT or a small cell phenotype, we crossed RASLO mice with the inducible keratin KO line (KII). RASLO mice with a wild-type keratin type II cluster (KII +/+) or harboring one wild-type and one knock-out allele (KII +/−) or one floxed and one knock-out allele (KII f/−) were treated with AdCre intranasally. All three groups of mice developed tumors that were detectable *in vivo* by bioluminescence imaging within 6 weeks (**Fig. 3A**). Six weeks after AdCre treatment, the mice were sacrificed and tumor burden and morphology was assessed. To confirm the genotype of the mice and the lung tumors, we performed PCR reactions on DNA extracted from whole lung of the mice with primers specific for the RASLO construct, the deleted and the floxed Keratin type two cluster (Fig. 3B). All mice showed the presence of RASLO construct, and as expected only KII +/− and KII f/− showed insertion of the DEL locus, and exclusively the mice with the KII f/− allele gave the expected PCR band (**Fig. 3B**). We used three different measures to quantify the tumor burden of KII +/+, KII +/− and KII f/− animals. Firstly, we quantified the tumor burden by measuring the amount of luciferase mRNA in tumor bearing lungs means of qRT-PCR (**Fig. 3C**). Luciferase expression can be correlated with tumor load as it is co-expressed by the RASLO construct. We did not observe a statistically significant difference in luciferase mRNA levels between the groups. This indicates that a reduced Keratin expression does not lead to an increased tumor load.

In addition we estimated the tumor load by assessing the % of lung area covered by tumor on three sagittal sections of the lungs by two blinded independent observers. This revealed that tumors of comparable size developed in the RASLO mice carrying the different keratin genotypes (**Fig. 3D**). In addition, tumor size was assessed by measuring the tumor diameter using images of scanned histological slides. A comparison between the different genotypes did not reveal any significant differences (**Fig. 3E**). These data suggest that the absence of at least one allele of the keratin type II cluster does not significantly impact the tumor biology in this KRAS-driven model of lung adenocarcinoma.

**Figure 4:**
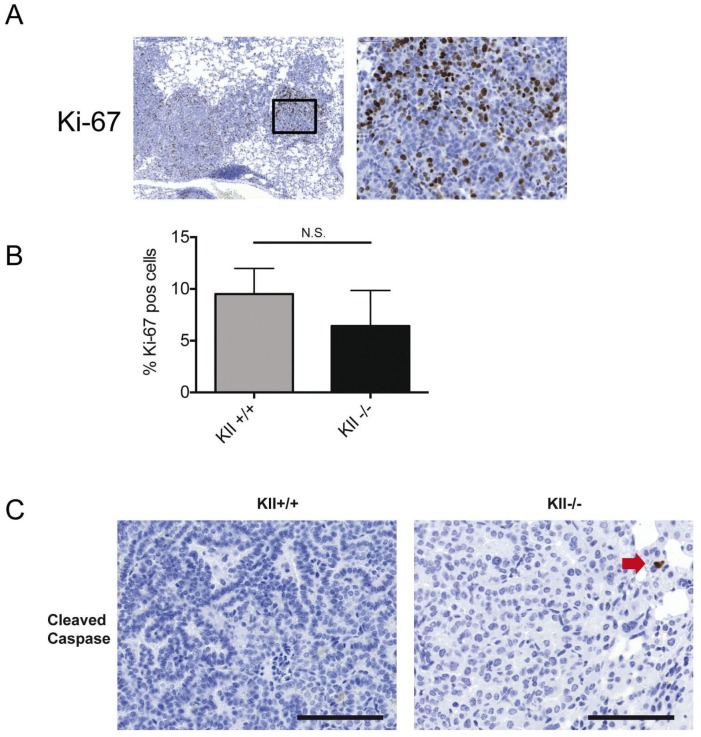
Keratin deficient and wild-type KRAS driven lung tumors have comparable rates of proliferation and apoptosis. (A) Ki-67 immunohistochemistry of a KII −/− deficient tumor (compare figure 5 from the same series of serial sections). (B) Formal analysis of 6 areas per lung of six Keratin wild-type and KII −/− tumors reveals similar Ki-67 proliferation index. Cleaved caspase 3 staining only showed single positive cells (red arrow) in in Keratin deficient (−/−) and Keratin expressing tumors (KII).

The activation of the KRAS construct requires the CRE-mediated removal of an 800 bp large DNA fragment including a STOP codon (**Fig. 1A**). To delete the entire type II keratin cluster, a DNA fragment of 0.68 Mbs needs to be removed [10], [22]. Therefore, we did not expect that all tumors in RASLO×KIIf/− mice would be completely devoid of keratins. To visualize those tumors in the lungs of RASLO×KIIf/− mice in which the floxed KII-allele was successfully removed, we stained paraffin sections for keratins 8/18. About 30% of tumors completely lacked keratin expression, indicating that indeed cre-mediated deletion of the KII cluster was not as efficient as the activation of the KRAS construct (**Fig. 3F**). Morphologically, the keratin-deficient tumors were typical papillary adenomas and adenocarcinomas indistinguishable from the neighboring keratin positive tumors on the same slide (**Fig. 3F**) or from KRAS-induced lung tumors from keratin wild type animals (**Fig. 3G**). To examine whether keratin loss had an influence on proliferation or apoptosis rate in KRAS driven lung tumors, we determined the proliferation rates and apoptosis rates by Ki67 and cleaved caspase 3 staining, respectively, but did not observe significant differences (**Fig. 4A, B**). In summary, these data provide evidence that loss of keratin expression does not affect tumor cell size of KRAS driven murine lung adenocarcinomas and that the presence of 30% keratin negative tumors did not alter overall tumor burden, suggesting that loss of keratin expression is not directly involved in the establishment of the small cell and aggressive phenotype of SCLC.

### Keratin Loss does not Influence the Expression of EMT and Neuroendocrine Markers in Lung Tumors

The reduction of keratin expression and condensation into a punctuate perinuclear pattern is a characteristic of neuroendocrine small cell lung cancer and is widely used as a marker for EMT in adenocarcinomas. Although the keratin negative tumors arising in the AdCre-treated KRAS×KIIf/− mice did not differ morphologically from their neighboring keratin positive tumors, we investigated whether the keratin negative tumors expressed additional markers associated with EMT and the SCLC phenotype.

**Figure 5:**
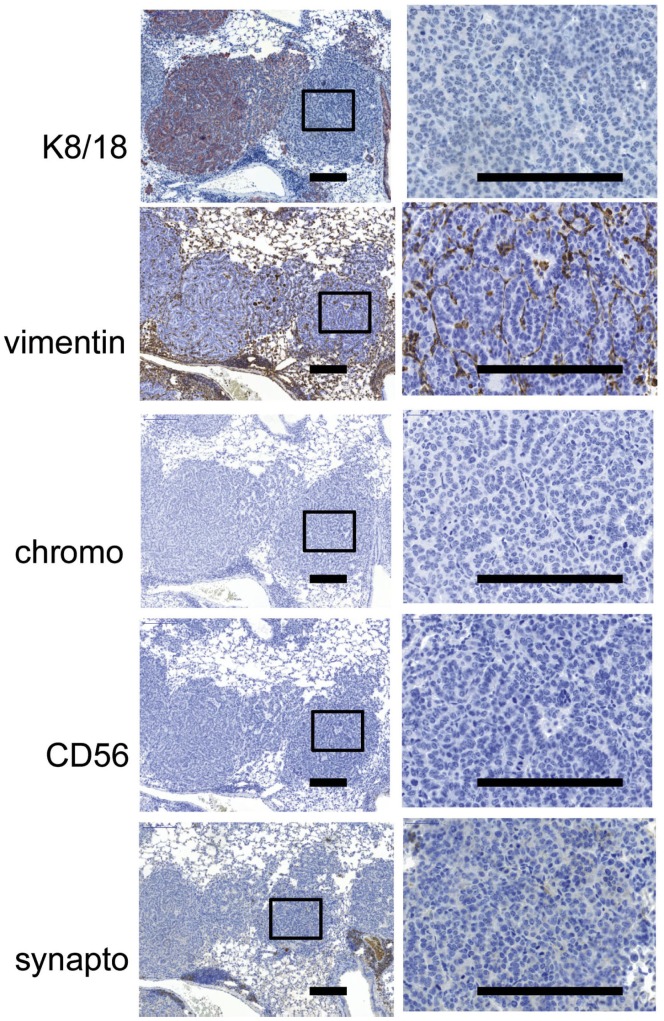
Loss of keratin expression in KRAS×KIIf/− tumors does not correlate with a small cell phenotype. Mice were anesthetized and 2×10^7^ PFU Adeno-CRE virus was pipetted on the nose of the mouse to be inhaled. Mice were sacrificed after 6 weeks and the lungs of the mice were removed and formalin fixed and paraffin embedded. IHCs for K8/18, vimentin, chromogranin A (chromo), CD56 and synaptophysin (synapto) are shown. A higher magnification of a Keratin negative tumor is shown in the right column and the area boxed on the left.

**Figure 6:**
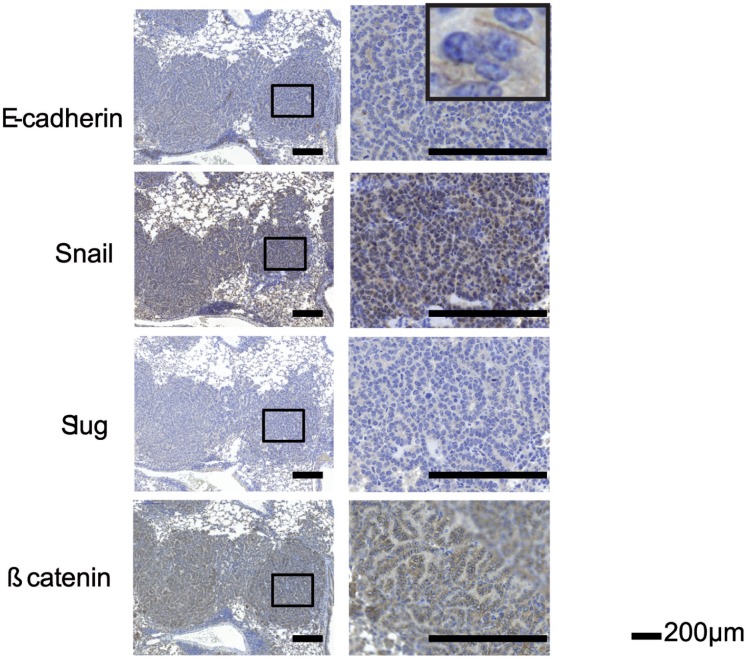
Loss of keratin expression in KRAS×KIIf/− tumors does not correlate with expression of EMT markers. Six weeks after Adeno-Cre administration, mice were sacrificed and the lungs of the mice were removed and formalin fixed and paraffin embedded. A higher magnification of a Keratin negative tumor is shown in the right column and the area boxed on the left. Stainings of serial section for EMT markers, i.e. E-cadherin, Snail, Slug, and β-catenin are shown.

Immunohistochemistry revealed that the type III intermediate filament protein vimentin, which is frequently up-regulated during epithelial to mesenchymal transition (EMT) after loss of keratin expression (Mani u. a., 2008), was neither expressed in keratin-positive nor -negative tumors of the KRAS×KIIf/− mice (**Fig. 5**). Furthermore, chromogranin A, CD56, and synaptophysin, are immunohistochemical markers typically expressed in small cell neuroendocrine carcinomas of the lung [23], [24]. However, both keratin-positive and -negative tumors in the KRAS×KIIf/− mice did not express any of these markers (**Fig. 5**). Positive controls for the specificity of stainings are shown in figure S1. We then analyzed markers associated with the loss of cell-cell contacts during EMT. In particular, loss of E-cadherin induced by several transcriptional repressors like Snail, and Slug [25] is typical for EMT. However, we did not find that keratin-deficient tumors over expressed the transcriptional repressors Snail, Slug or lost E-cadherin expression **(** **Fig. 6** **)**.

Development and maintenance of adherens junctions depends on a multiprotein complex of β-catenin, α-catenin and E-cadherin. Upon activation of Wnt signaling, a considerable proportion of ß-catenin in being translocated into the nucleus to switch on gene expression associated with EMT [26]. We stained for ß-catenin but could not detect any nuclear ß-catenin expression in keratin wild-type or deficient tumors (**Fig. 6**). We have previously shown in cell lines that lack the KII cluster, ZO1 and Desmoplakin are no longer located at the membrane [22] We have attempted to stain for Desmoplakin and ZO1 on murine FFPE material. In Figure S2 the staining of wildtype murine skin and KII +/+ for ZO1 are shown. We could detect some membranous staining in the skin sample that served as a positive control. However, we did not see any membranous staining in the wild type or KII−/− tumors. In addition, we stained RASLO wild-typ and keratin deficient tumors for pMAPK and CD44 and did not observe and difference (Fig. S2). In summary, keratin loss in our KRAS lung tumor model did not lead to the development of tumors with a small cell phenotype or expression of markers associated with EMT. These data indicate that keratin loss by itself is not responsible for the onset of EMT or the development of a small cell phenotype in adenocarcinomas of the lung in the mouse model presented here.

## Discussion

Loss of keratin expression is a hallmark of both small cell lung cancer and EMT and has been suggested to enhance invasion and metastasis. Furthermore, in adenocarcinomas of the lung which have been treated with novel EGFR tyrosine kinase inhibitors, conversion to a small cell phenotype and EMT has been identified as one of several resistance mechanisms to evade therapy [8].

In order to investigate whether keratin loss in adenocarcinomas confers the phenotypic characteristics of EMT or a small cell phenotype we crossed inducible keratin-deficient mice with our newly developed RASLO mice harboring an inducible mutated KRAS^Val12^.

We here show that intranasal application of an adenovirus expressing CRE-recombinase was sufficient to induce expression of a mutated KRAS^Val12^ leading to the development of tumors in the lung (**Fig. 1**), but not in any other tissues. These tumors range from papillary adenomas to invasive papillary adenocarcinomas, which is consistent with KRAS-driven lung cancer models described previously [4], [20]. The lung tumors developed rapidly within 4–6 weeks, which holds an advantage over several other mouse models where tumor formation progresses over months [27]. The strong chicken ß-actin promoter that drives the expression of a mutated KRAS^Val12^ may be responsible for the rapid tumor onset in this model. Moreover, we were able to monitor tumor formation non-invasively by bioluminescence imaging due to the simultaneous expression of luciferase and KRAS^Val12^ from the same promoter, which is an improvement compared to models that rely on luciferase expression by a separate, second recombination event, potentially leading to the development of luciferase negative tumors [28].

After AdCre-mediated tumor induction in RASLO×KIIf/−, we successfully generated mice harboring both keratin-proficient and –deficient adenocarcinomas growing side-by-side in the same lung. However, we found no evidence that complete loss of keratin expression in any way promoted EMT or the emergence of small cell phenotype tumors in the lung (**Fig. 2** **,** **3** **,** **4** **,** **5** **,** **6**). Overall tumor burden in RASLO×KIIf/− mice was not different compared to RASLO×KII+/− or RASLO KII+/+ mice. Tumor morphology and cell size were similar and proliferation rates in keratin wild-type and deficient tumors were not changed. Moreover, markers associated with EMT and progressive disease, were not induced in keratin-deficient tumors. Taken together, these data support the notion that even complete loss of keratin expression does not drive EMT and is not causally promoting the acquisition of a small cell phenotype.

Interestingly, although some studies support an active role of keratins in cell invasion and metastasis formation [29], keratin knock-out mice [10] did not develop tumors at an increased rate or metastatic capacity. Furthermore, keratin 10 knock-out mice displayed reduced tumor formation in a 2-stage skin carcinogenesis model, possibly caused by an accelerated turnover of keratinocytes due to activation of MAPK pathways [30]. Our data are consistent with these findings. On the contrary, co-expression of keratin 8 and 18 together with vimentin leads to elevated invasion and cell migration in human melanoma [31]. The acquired expression of vimentin in keratin-deficient tumor cells undergoing EMT may function as a compensatory mechanism to maintain cell integrity or may result from an activation of the vimentin promoter in the tumor setting. The keratin-negative tumors in the RASLO×KIIf/− mice did not gain expression of vimentin and were neither more invasive nor prone to metastasize than their keratin-positive counterparts. From these data, one could speculate that gain of vimentin expression additionally to keratin loss in NSCLC may be a more indicative marker for the development of a more aggressive tumor than keratin loss alone.

Finally, we found that expression of the known regulators of EMT Slug and Snail was not induced and ß-catenin expression not altered in keratin-deficient tumors. This implies that the activation of signaling pathways leading to EMT is not under downstream control of a functional keratin cytoskeleton, at least in this model, and hence, changes in keratin expression and cytoskeletal reorganization are secondary phenotypic events that have no or little effect on tumor biology in KRAS-driven lung cancer.

Thus, although keratin loss is characteristic for tumors that have undergone EMT or have gained a small cell phenotype, we propose that the changes in cell shape and migration that underlie EMT and the small cell phenotype are not causally determined by changes or loss of keratin expression, based on our novel mouse model.

## Supporting Information

Figure S1 **Control staining for IHCs shown in** **Figure 5** **and** **6** **.** Positive controls for the different antibodies are shown. A murine small cell lung cancer cell line derived from a p53 and RB1 knock-out mouse was used for CD56 staining, adult murine brain tissue was used for synaptophysin stainig, adult murine adrenal gland was used for Chromogranin A, and fetal cartilage for Snail and Slug staining. The scale bar indicates 50 µm.
(EPS)

Figure S2 Loss of keratin expression does not lead to up regulation of pMAPK or CD44 (A) and not changes in ZO1 expression (B). (A) Formalin fixed and paraffin embedded tumors of RASLO mice with wild-type keratin expression (KII +/+) or deficient for the keratin type two cluster (KII−/−) show no significant difference in pMAPK or CD44 expression. (B) ZO1 staining is barely detectable on FFPE skin samples of wild type mice, but not detectable in wild type (KII+/+) or Keratin deficient (not shown) RASLO tumors.
(EPS)
